# Supplementary material for: Oleanolic Acid Complexation with Cyclodextrins Improves Its Cell Bio-Availability and Biological Activities for Cell Migration
Source: Int J Mol Sci. 2023 Oct 3;24(19):14860. doi: 10.3390/ijms241914860 (PMC10573973; doi:10.3390/ijms241914860)
Supplement: Supplementary file 1 [file ijms-24-14860-s001.zip › ijms-2626750 - SM/ijms-2626750-supplementary-figure lend.docx]

Figure S1. Non‑dried OA/CDs complexes have poor effectivity on cell migration as opposed to freeze dried OA/CDs complexes in Mv1Lu cells. Confluent Mv1Lu cells were scratched with a pipette tip and allowed to migrate for 24 hours. (a) Plot represents cell migration as the difference between areas at time 0 hours and time 24 hours in each condition, named as migration percentage. X axis indicates treatment conditions, for those with HP‑γ‑CD, the concentrations are represented as molar ratio OA μM /CD mM. Non‑dried OA/HP‑γ‑CD complexes concentrations used are represented in grey bars and white triangles. Freeze dried 50/12.5 μM/mM OA/HP‑γ‑CD complexes and 5 µM OA/DMSO were used as positive control (black bars). Empty HP‑γ‑CDs at 0/12.5 μM/mM were used as vehicle control. Epidermal growth factor (EGF) was added at 10 ng/ml as a positive migration control. Asterisks indicate statistically significant differences between conditions according to a One‑way ANOVA statistical analysis (****p<0.0001). (b) Representative images of the wound healing assay with cell migration under 5 μM OA/DMSO compared to those non‑dried OA/HP‑γ‑CD complexes after 24 h treatment. DMSO, and 0/12.5 μM/mM HP‑γ‑CD were used as vehicle controls.

Figure S2. OA complexes with modified cyclodextrins HP‑β‑CD and HP‑γ‑CD promote cell migration in scratched Mv1Lu cells. Plot represents cell migration as the difference between areas at time 0 hours and time 24 hours in each condition, named as migration percentage. X axis indicates treatment conditions, for those with HP‑β‑CD and HP‑γ‑CD, the concentrations are represented as molar ratio OA µM/CD mM. Additional concentrations of OA/CDs are shown. Note that concentrations without any data point indicate cell viability loss as a consequence of the OA cytotoxic effect at high OA concentrations. 10 % FBS (S 10%) and 10 ng/ml EGF were also added to the experiment as positive migration controls. Asterisks indicate statistically significant differences between conditions according to a One‑way ANOVA statistical analysis (*p<0.05, **p<0.005, ***p<0.001 and ****p<0.0001).

Figure S3. OA complexes recruit a higher number of Mv1Lu migratory‑cells than OA/DMSO on in vitro scratch assays. (a) Composite pictures of the in vitro scratch assay showing the first lines of cells at the scratch edge and cells far away from the edge. Images show cell migration under basal conditions (Control) compared to those with 5 µM OA/DMSO and 12.5/3.12 µM/mM OA/HP‑β‑CD after 24 hours treatment. Note that with OA/HP‑β‑CD complexes a greater number of cell lines are recruited to migrate along the scratch. (b) Pictures corresponding to the same assay showing vehicle control conditions with equivalent concentrations. Scale bar 200 µM.

Figure S4. OA/HP β CD complexes promote higher c Jun transcription factor phosphorylation than OA/DMSO at the scratch edge. (a) The image represents how a Tile Scan image is divided in three equal sectors for better analyze active c Jun (p c Jun) expression. Scale bar indicates 100 µm. (b) Plot represents the p c Jun intensity in cell nuclei. Each point represents the intensity of p c Jun intensity in one nucleus. (c) Plot represents the relation between the number of positive p c Jun nuclei and total number nuclei existent in each sector. In order to exclude negative p c Jun nuclei, an intensity p c Jun threshold was set using the p c Jun intensity mean in basal (control) condition.

Figure S5. OA/HP β CD complexes change the distribution of actin cytoskeleton, revealed by Phalloidin staining. Confluent Mv1Lu cells were scratched and allowed to migrate for 6 and 12 hours. Cells were treated with 5 µM OA/DMSO or 12.5/3.12 µM/mM OA/HP β CD. Equivalent concentrations of DMSO and HP β CD were used as vehicle controls. Detailed images of the staining showed in Figure 4, corresponding to the cells at the scratch edge (sector 1, S1, area) and their 2 times magnified homologue. Actin fibers (F Actin): red. Nuclei: blue. Images obtained with a confocal microscope at 40X. Sector 1 scale bar indicates 50 µm. Magnified Sector 1 scale bar indicates 25 µm.

Figure S6. OA/HP β CD complexes promote changes in focal adhesions (FAs) revealed by Paxillin. Confluent Mv1Lu cells were scratched and allowed to migrate for 6 and 12 hours. Cells were treated with 5 µM OA/DMSO or 12.5/3.12 µM/mM OA/HP β CD. Equivalent concentrations of DMSO and HP β CD were used as vehicle controls. Cells were immunostained with specific antibodies against Paxillin. Co-staining with phalloidin and Hoechst 33258 was used to show actin cytoskeleton and nuclei, respectively. Paxillin: green. Actin fibers (F Actin): red. Nuclei: blue. Images obtained with a confocal microscope at 40X. This experiment was repeated at least three times. Scale bar indicates 25 µm.

Figure S7. Cell migration necessary signaling pathways regulated by EGFR and c‑Jun activation, are induced by OA/HP‑β‑CD complexes in Mv1Lu cells. (a) Total protein extracts from sub-confluent Mv1Lu cells in FBS‑free media treated with 10 µM OA/DMSO or 12.5/3.12 µM/mM. DMSO and HP‑β‑CD equivalent concentrations were added as vehicle controls. Different proteins were assayed at the indicated times (hours): phospho‑EGFR (Tur 1068), phospho‑ERK1/2 (Thr 202/Tyr 204), phospho‑JNK1/2 (Thr 183/Tyr 185) and phospho‑c‑Jun (Ser 63). Total protein expression was assayed for the above‑mentioned active forms: ERK1/2, JNK1/2 and c‑Jun. β‑Actin was used as a loading control. A representative experiment was shown (EGFR, epidermal growth factor receptor; ERK1/2, extracellular signal‑regulated kinases 1 and 2; c‑Jun N‑terminal kinases 1 and 2). (b) Column bar graphs represent intensity values of each protein assayed by Western blot, by collecting the data of three independent experiments. Intensity values were quantified and gathered by ImageJ software. Asterisks indicate statistically significant differences between the selected conditions according to a One‑way ANOVA statistical analysis: (*p<0.05, **p<0.005, ***p<0.001 and ****p<0.0001).

Figure S8. Full-length blots corresponding to crops showed in Fig 6. (a) Tyr 1068 Phosphorylated-EGFR. (b) EGFR. (c) Thr 202/Tyr 204 Phosphorylated ERK (d) ERK1/2. (e) Thr 183/Tyr 185 Phosphorylated JNK. (f) JNK1/2. (g) c-Jun. (h) Ser 63 Phosphorylated c-Jun. (i) Beta-actin loading. Dashed red rectangle indicates the portion of the blot that was used in the figure.

Figure S9. Full-length blots corresponding to crops showed in Supplemental Fig 7. (a) Tyr 1068 Phosphorylated-EGFR. (b) Thr 202/Tyr 204 Phosphorylated ERK. (c) ERK1/2. (d) c-Jun. (e) Thr 183/Tyr 185 Phosphorylated JNK. (f) JNK1/2. (g) Ser 63 Phosphorylated c-Jun. (h) Beta-actin loading. Dashed red rectangle indicates the portion of the blot that was used in the figure.
