# Supplementary figures and images for: Oleanolic Acid Complexation with Cyclodextrins Improves Its Cell Bio-Availability and Biological Activities for Cell Migration
Source: Int J Mol Sci. 2023 Oct 3;24(19):14860. doi: 10.3390/ijms241914860 (PMC10573973; doi:10.3390/ijms241914860)

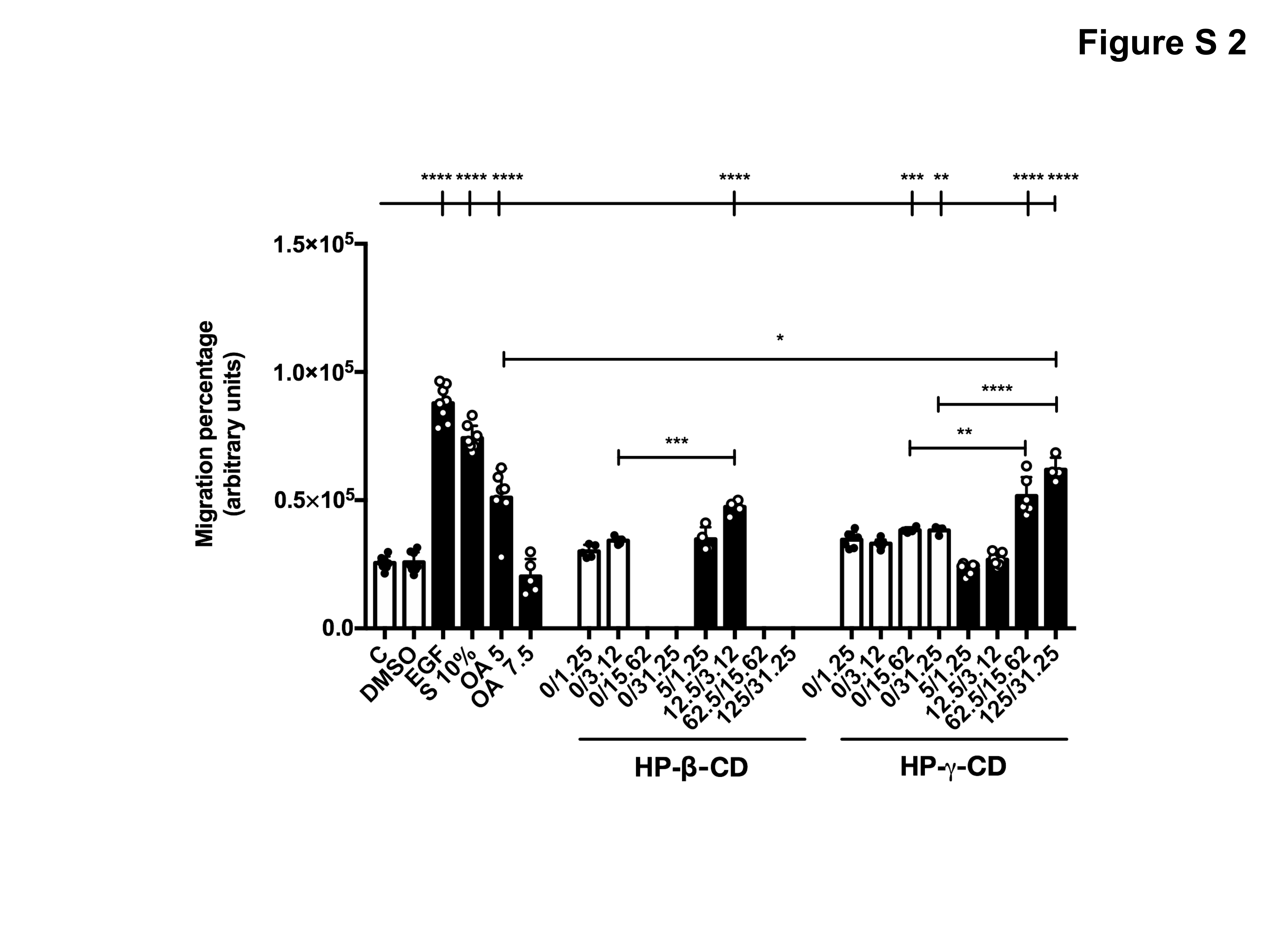

Supplement: Supplementary file 1 [file ijms-24-14860-s001.zip › ijms-2626750 - SM/Fig S 2.tif]

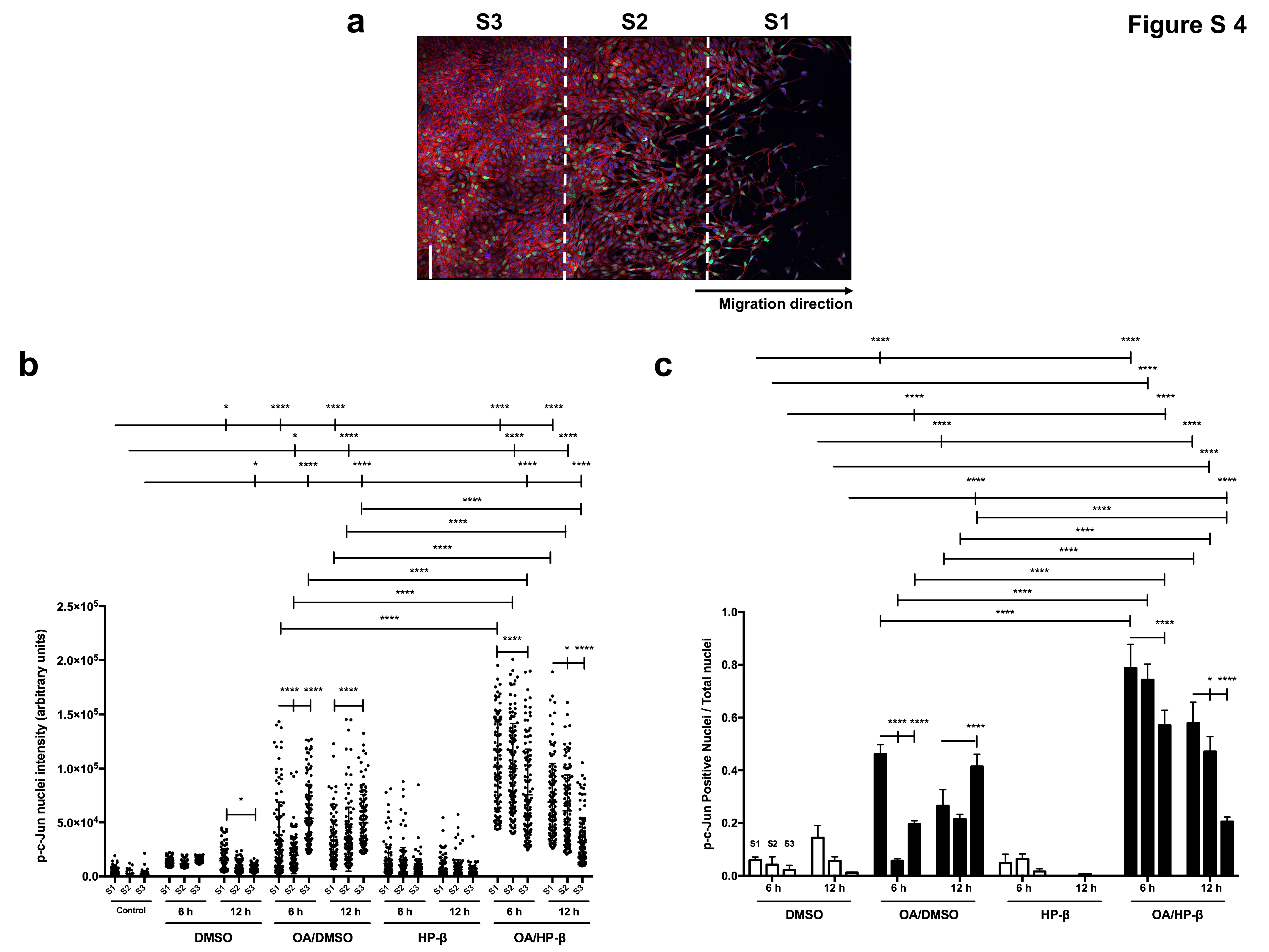

Supplement: Supplementary file 1 [file ijms-24-14860-s001.zip › ijms-2626750 - SM/Fig S 4.tif]

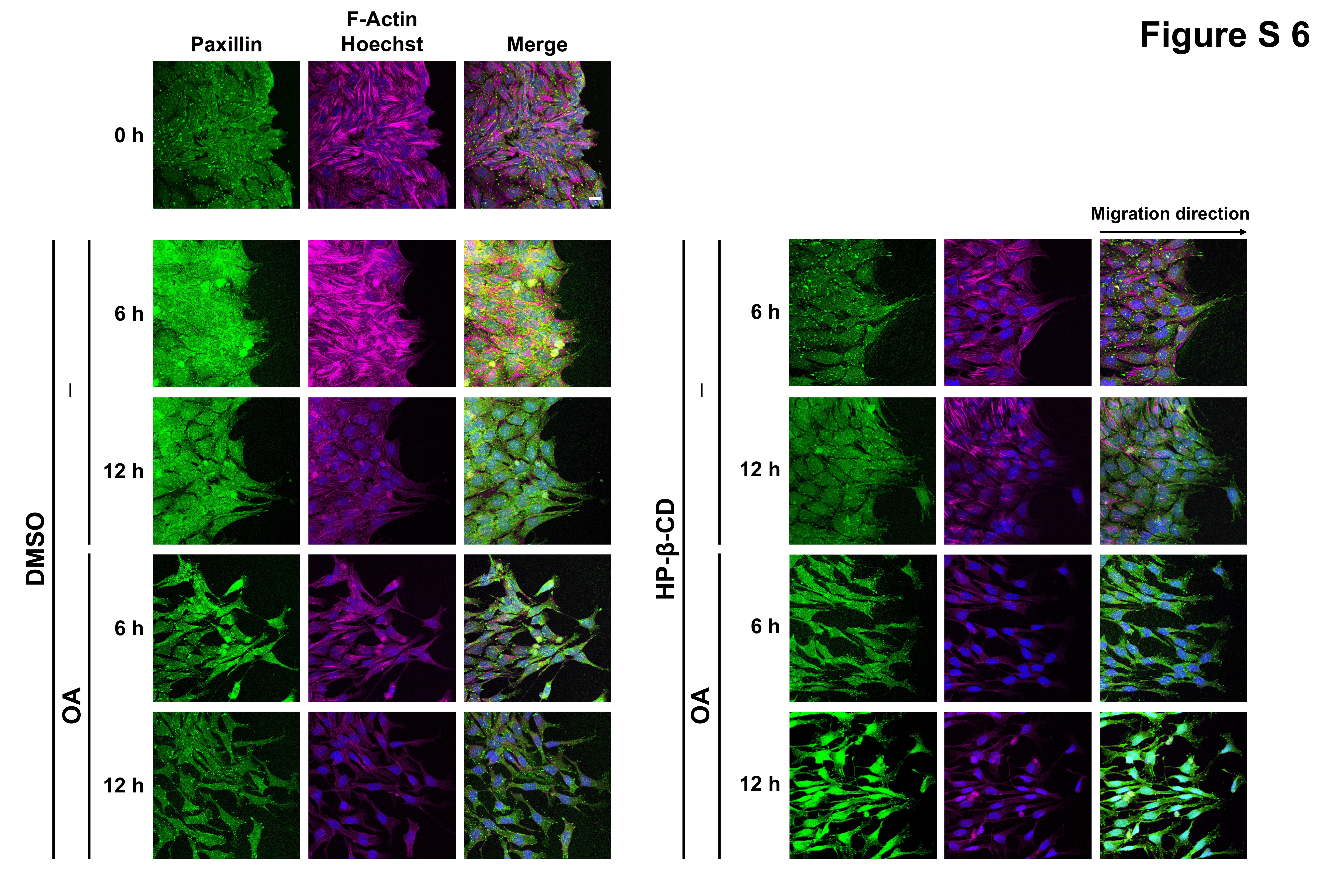

Supplement: Supplementary file 1 [file ijms-24-14860-s001.zip › ijms-2626750 - SM/Fig S 6.tif]

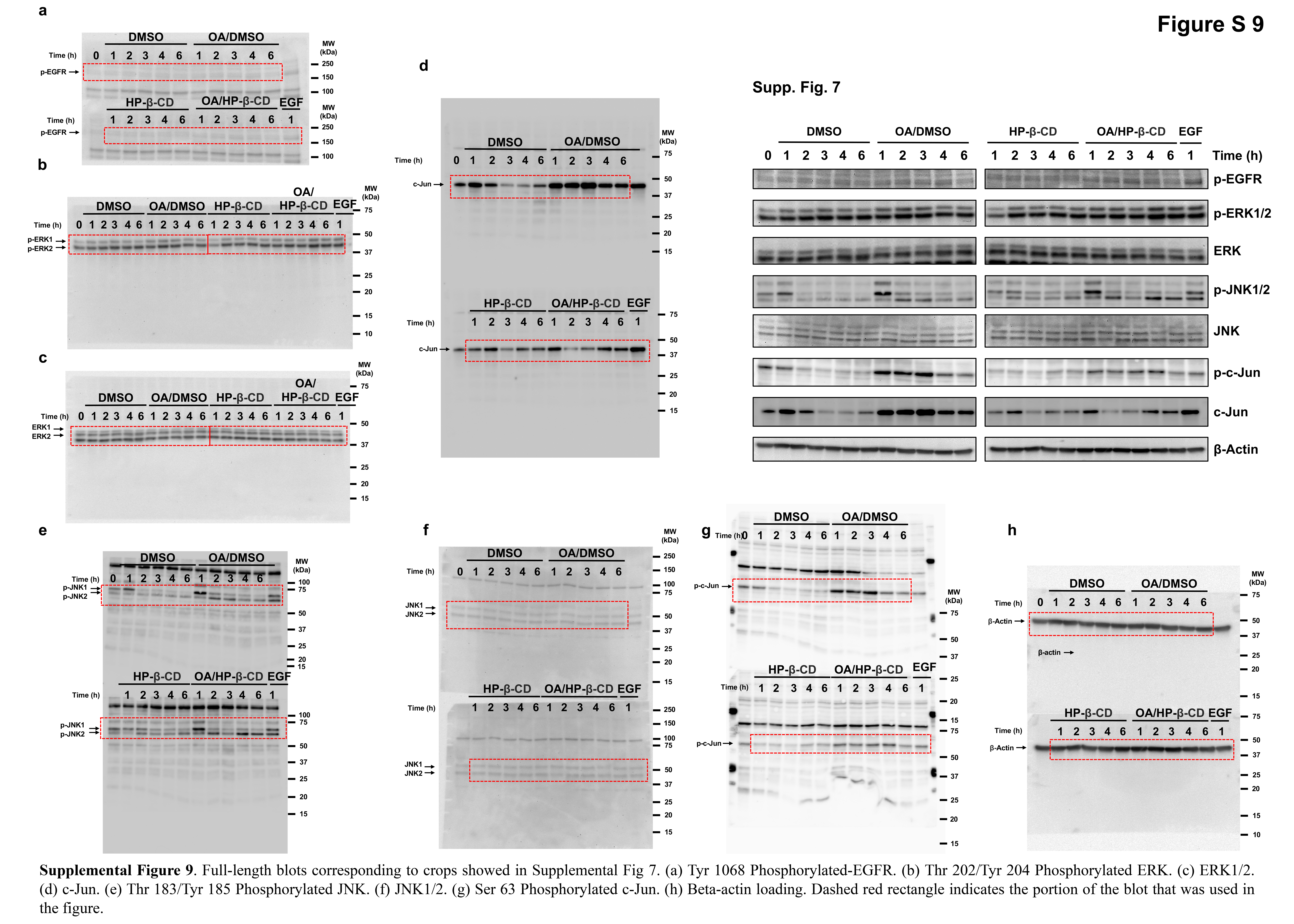

Supplement: Supplementary file 1 [file ijms-24-14860-s001.zip › ijms-2626750 - SM/Fig S 9.tif]
